# Passive Articulated and Non-Articulated Ankle–Foot Orthoses for Gait Rehabilitation: A Narrative Review

**DOI:** 10.3390/healthcare11070947

**Published:** 2023-03-24

**Authors:** Hasan Mhd Nazha, Szabolcs Szávai, Mhd Ayham Darwich, Daniel Juhre

**Affiliations:** 1 Faculty of Mechanical Engineering, Institute of Mechanics, Otto Von Guericke University Magdeburg, Universitätsplatz 2, 39106 Magdeburg, Germany; 2 Faculty of Mechanical Engineering and Informatics, University of Miskolc, 3515 Miskolc, Hungary; 3 Faculty of Biomedical Engineering, Al-Andalus University for Medical Sciences, Tartous, Syria

**Keywords:** rehabilitation, ankle–foot orthosis, foot drop, passive AFO, articulated AFO, non-articulated AFO

## Abstract

The aim of this work was to study the different types of passive articulated and non-articulated ankle–foot orthoses for gait rehabilitation in terms of working principles, control mechanisms, features, and limitations, along with the recent clinical trials on AFOs. An additional aim was to categorize them to help engineers and orthotists to develop novel designs based on this research. Based on selected keywords and their composition, a search was performed on the ISI Web of Knowledge, Google Scholar, Scopus, and PubMed databases from 1990 to 2022. Forty-two studies met the eligibility criteria, which highlighted the commonly used types and recent development of passive articulated and non-articulated ankle–foot orthoses for foot drop. Orthotists and engineers may benefit from the information obtained from this review article by enhancing their understanding of the challenges in developing an AFO that meets all the requirements in terms of ease of use, freedom of movement, and high performance at a relatively low cost.

## 1. Introduction

Human walking is typically conceptualized as the repetitive movement of the limbs and is a distinctive feature of human locomotion, with each individual possessing a distinct style of walking [1]. Humans with conditions that affect the hip, knee, or ankle bones, nerves, muscles, or joints may have difficulties in walking. This can lead to falls and injuries if the problems are not addressed [2]. Deficiencies in the nerves, muscles, bones, or spinal cord can also cause people to have trouble walking; these deficiencies are typically hereditary. People with gait disabilities can be aided by devices such as assistive footwear or rehabilitation. This process is referred to as gait rehabilitation and typically involves several stages [3,4]. Ankle-foot orthoses are used to support patients with abnormal gait; they are also used for rehabilitation [5]. These orthotics devices are typically referred to as ankle–foot orthoses or AFOs. Passive AFOs do not contain electrical boards, but they incorporate springs, dampers, or mechanisms that control the motion between the bone stubs in the foot [6]. Passive AFOs can be used in daily life, as they are compact in size [5].

There are different classifications for passive AFOs. For example, based on the relative motion between the shank parts of AFOs and the foot [7], they are also classified into two types: articulated and non-articulated (fixed) AFOs. While articulated AFOs are two-piece devices made of lightweight thermoplastics or carbon composites connected by joints, non-articulated AFOs are single pieces made of lightweight thermoformable materials, such as polyethylene or polypropylene. The shank parts of the AFO and the foot are connected by using springs, dampers, hinges, or flexion stops [8]. According to Alexander and Xing [9] other classifications are used to define AFO types. Solid AFO (SAFO) refers to solid plastic AFOs (PAFOs) or metallic AFOs [10], rigid AFOs [11,12], fixed AFOs [13], or solid AFOs [14,15,16,17,18]. Hinged AFO (HAFO) refers to hinged AFOs [15,16,17,19,20,21] or articulated AFOs [10,12]. Posterior -leaf-spring AFO (PLS) refers to posterior-leaf-spring and spring-type AFOs [17,22,23]. Floor-reaction AFO (FRO) refers to FROs [24,25] or ground-reaction AFOs [10,26]. Finally, carbon-fiber-spring AFOs (CFOs) have been described [13]. According to [27], the term passive AFO comprises only six categories: posterior-leaf-spring AFO, solid AFO, short-Leg AFO, dorsiflexion-assist AFO, plantar-flexion-stop AFO, and energy-return AFO. However, this paper studies the different types of passive articulated and non-articulated ankle–foot orthoses for gait rehabilitation in terms of working principles, control mechanisms, features, and limitations, along with the recent clinical trials on AFOs. In addition, this study categorizes AFOs to help engineers and orthotists to develop novel designs based on the literature.

## 2. Methods

**Figure 1:**
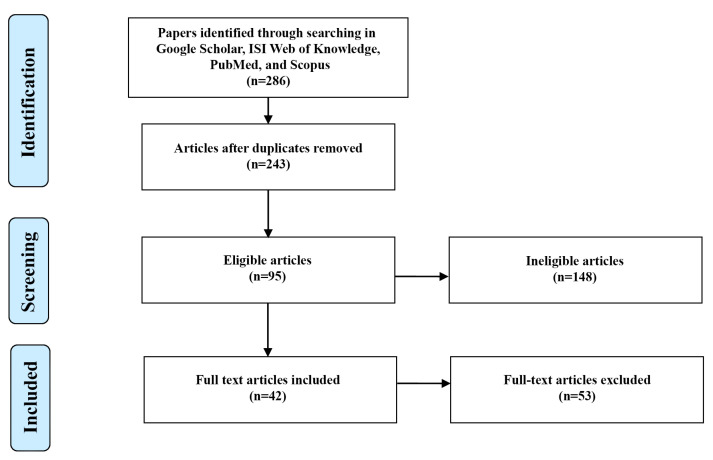
Selection procedure utilized in this study.

A literature search was conducted on Scopus, Google Scholar, ISI Web of Knowledge, and PubMed (from 1990 to 2022), as shown in Figure 1, and cited references from proper articles were thoroughly reviewed. The selected keywords in the search process were: “ankle-foot orthosis (AFO)”, “passive”, “articulated”, “non-articulated”, “solid AFO (SAFO)”, “posterior leaf spring AFO (PLS)”, “floor reaction AFO (FRO)”, “carbon fiber AFO (CFO)”, “short leg AFO”, “dorsiflexion assist AFO”, “plantarflexion stop AFO”, “energy return AFO”, “3D printed AFO”, and “SMA AFO”.

By adopting the comparative research equation, the main research question devised was: What are the comparative features and limitations of different types of passive articulated ankle–foot orthoses for gait rehabilitation? Inclusion criteria: Studies that investigated the use of passive articulated and non-articulated ankle–foot orthoses for gait rehabilitation, studies that compared different types of passive articulated and non-articulated ankle–foot orthoses, studies published in peer-reviewed journals between 1990 and 2022, and studies that were written in English or for which English translations were available. Exclusion criteria: Studies that investigated the effects of ankle–foot orthoses on athletic performance, rather than gait rehabilitation; studies that were based on animal models or in vitro testing, rather than human subjects; studies published before the year 1990, or not published in peer-reviewed journals; and studies that were not written in English or did not have English translations available.

## 3. Results and Discussion

### 3.1. Metal AFOs

**Figure 2:**
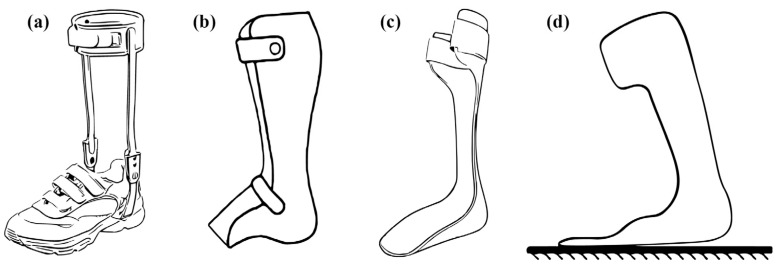
Solid AFOs: (**a**) metal AFO, (**b**) plastic AFO, (**c**) posterior-leaf-spring AFO, (**d**) Ground-reaction AFOs.

Metal AFOs (conventional AFOs) provide support for the foot, ankle, and lower leg [28]. They are used by patients with damaged nerves and muscles in their lower extremities [29]. These prostheses provide support through a calf strap, which creates direct skin-to-skin contact (Figure 2). Metal ankle supports connect to the shoes via bars that do not touch the wearer’s skin; they reduce pressure on the soft tissue in the ankle by distributing weight across several ankle fins. However, their heavy weight and cumbersome nature are among their major drawbacks. Therefore, users currently find it practical to replace these metal supports with a more energy-efficient alternative: plastic AFOs [30]. Berkelman et al. [31] presented a four-bar mechanism used in an ankle–foot-orthosis design. This design had link joints that were pivoted with aluminum bars.

A portion of this design involves providing an additional force to aid in lifting the foot during the swing phase. The concept behind this device is that the knee and ankle move together to connect and generate this assistive force. A curved bar attaches at the calf and foot, along with a link attachment that connects it to the back of the thigh. When the knee is flexed at 5 to 20 degrees, the thigh makes contact with the bar to generate a lifting action through the four-bar linkage motion at the ankle. A knee that is not bent provides additional torque to the ankle when it is pushed into a flexed position. However, keeping the knee straight prevents this force from being generated. Instead, increased torque at the ankle occurs through knee flexion. The main drawbacks of this prototype include its bulky appearance, lack of toe-drag assistance, uncomfortable weight distribution, and lack of foot-slap prevention. Additional adjustments can be made to the timing and amount of assistance by changing the spring stiffness, the lengths of the links, and the point of attachment. In some cases, individual users can adjust these factors. Ghosh et al. [32,33] introduced an AFO containing a six-bar mechanical linkage. The study also established 11 task points (standard data) for the synthesis of toe movement during normal walking. Upon evaluation, it was found that there was a ±10 degree of discrepancy between the simulation data of the device and the standard data of a healthy individual. A gait analysis revealed certain limitations in this AFO, including a ±12 percent error in the knee and ankle angles and one missing task point for the toe movement out of the eleven task points defined for normal walking.

### 3.2. Plastic AFOs (PAFOs)

Plastic AFOs (PAFOs) are mainly made of thermoplastics, such as polyethylene or polypropylene, and are among the most widely used solid orthoses in clinical practice due to their numerous advantages, such as their relatively low cost, the ease with which they can be cleaned, good aesthetics, and easy desorption [34,35]. Plastic AFOs (Figure 2b) can be utilized to limit motion in the sagittal plane of the ankle during stance and swing phases, as well as to provide medial and lateral stability during different stances and anterior entry, with the use of support strapping at the proximal end and, potentially, at the ankle and forefoot. The underlying mechanism of these devices is the provision of the force necessary to generate an ankle plantar-flexor moment, thus enabling weight bearing on the distal aspect of the foot.

The stiffness of PAFOs depends on the shape of the flexible region, material properties, and thickness. However, their fabrication is carried out by the trial-and-error method, resulting in a negative effect on the knee during walking. If these PAFOs are overly stiff, they may delay the loading response, and the knee can become more flexed. If the PAFOs are less stiff, they affect the patient through the excessive extension of their knee. Therefore, PAFOs should be designed to provide minimum ankle stiffness [36].

### 3.3. Posterior-Leaf-Spring AFOs (PLS AFOs)

Posterior-leaf-spring AFOs (PLS AFOs) are solid AFOs (SAFOs), but, unlike conventional AFOS [37], they have a characteristic trim line located behind the ankle and leaf-shaped corrugation near the ankle (Figure 2c). The leaf-like creases are intended to strengthen the part of the ankle with the greatest amount of movement and repeated loadings. In addition, they act as a spring in the ankle, which allows slight dorsiflexion in the mid and terminal stances, and this elasticity can also marginally assist the push-off function in the terminal stance [38].

Furthermore, PLS AFOs can be applied to limit excessive equinus during swinging, thinner ankle coverage, which allows sagittal-plane motion in dorsiflexion during weight-bearing, a trim line posterior to the medial and lateral malleolus, and support strapping at the proximal tibia. In addition, they increase control over the instability of the ankle, as the ankle trim line extends further to the front of the ankle joint. However, PLS AFOs do not contribute significantly to ankle stability, as the trim line is behind the ankle. Thus, PLS AFOs are limited in their control of the varus/valgus [38,39].

### 3.4. Ground (Floor)-Reaction AFOs (GRAFOs or FRAFOs)

Ground- or floor-reaction ankle–foot orthoses (GRAFOs or FRAFOs) are types of custom-fabricated, molded plastic, AFO capable of tri-planar control of the foot/ankle complex [26]. Ground-reaction ankle–foot orthoses (Figure 2d) can be applied to provide ankle support to reduce ankle dorsiflexion and excessive knee flexion in stance via plantar-flexion knee-extension couple, rear or anterior entry, and large proximal tibial strapping, to support greater forces on the anterior tibia. Furthermore, they can be used in cases of adult-acquired flatfoot, posterior tibial tendon dysfunction (PTTD), cerebral palsy, brain injuries, Achilles tendonitis, osteoarthritis, spina bifida, spinal cord injuries, and post-polio paralysis.

Furthermore, SAFOs and GRAFOs are types of AFO that both apply a corrective internal plantar-flexion momentum to the ankle. Both apply this correction by producing an ankle-dorsiflexion moment through similar mechanical means [40]. In functional terms, they are similar. The GRAFOs use an anterior tibial shell to connect to the tibia in order to prevent the dorsiflexion of the ankle. Other GRAFO factors include using particular values, such as resistance to ankle flexion. The design of AFOs, despite significant variation, has only two basic types. One is the tibial shell design, and the other involves the use of a neutral angle, which alleviates the effects of knee flexion. It has been shown that GRAFO designs do not outperform SAFO designs in terms of reducing excessive knee flexion for individuals with certain disorders, such as CP. Instead, it has been shown that SAFOs are more effective at reducing knee flexion for individuals with CP, and that they have a significant effect on improving crouch gait for people with disabilities.

### 3.5. Type I, Type II, and DACS AFOs

**Figure 3:**
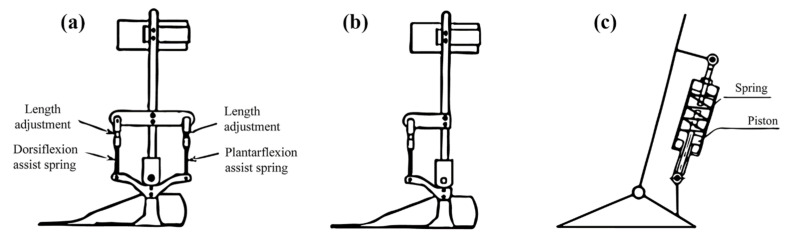
(**a**) Type I AFO, (**b**) Type II AFO, (**c**) DACS AFO.

Yamamoto et al. [41] introduced AFOs for individuals with hemiplegia, in which the level of assistive moments and the initial angle of the ankle can be adjusted easily. The design of these AFOs, referred to as Type 1 AFOs, included both dorsiflexion- and plantar-flexion-assistance moments. The foot component of the AFO was constructed by plastic material connected by two aluminum uprights with Klenzak joints and featured two springs, one of which was located on the anterior and another on the posterior side of the ankle joint (Figure 3a). In contrast, the anterior spring generates the dorsiflexion-assistance moment, while the posterior spring generates the plantar-flexion-assistance moment [42]. These assistance moments and the initial angle of the ankle can be separately adjusted by altering the lengths of the springs. As a result of their gait analysis, the authors determined that the presence of the posterior spring caused discomfort for hemiplegic patients and, therefore, a plantar-flexion assistance moment was deemed unnecessary for this patient population. Consequently, Type 2 AFOs (Figure 3b) were developed, which do not incorporate a posterior spring. This design allows greater freedom of movement in the ankle during dorsiflexion. A gait analysis revealed that this design leads to a reduction in the knee-flexion moment and the absence of quick plantar flexion in the ankle [41,42,43].

To provide assistance to people with DACS AFOs, a spring-loaded mechanism was added to the device’s shank. Due to these innovations, DACS AFOs were able to help people with restricted mobility. These AFOs contained two pieces: a plastic foot and an aluminum shank connected by an ankle (Figure 3c). By lengthening the assistance device, the DACS AFO’s ankle angle could be altered [6]. The application of a piston to compress the spring in the AFO design resulted in the generation of an assistance moment that was proportional to the plantar-flexion angle when the ankle rotated towards plantar flexion. However, when the ankle rotated in the opposite direction, towards dorsiflexion, the foot component of the AFO was able to rotate freely due to the minimal friction in the slider component and, thus, did not generate a plantar-flexion-assistance moment. The implementation of this DACS AFO design was found to enhance walking speed through the reduction in knee hypertension and the improvement in delayed progression in the hip joint. However, a drawback of this design was the utilization of large spring units, which contributed to its bulky size [6].

### 3.6. Plantar-Flexion-Stop AFOs (AFO-PSs)

**Figure 4:**
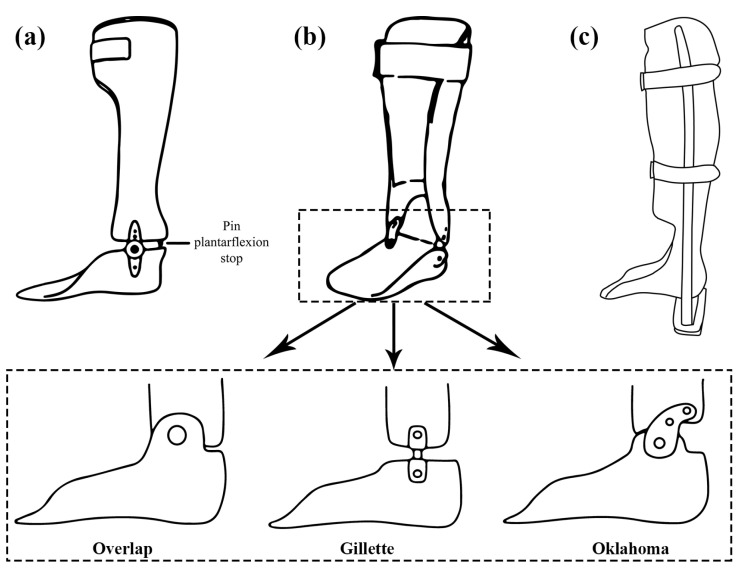
(**a**) Plantar-flexion stop AFO, (**b**) hinged AFO, (**c**) patellar-tendon-bearing AFO.

An investigation was conducted to determine the impact of the plantar-flexion resistance of ankle–foot orthoses (AFO-PS) on the gait of stroke patients in the subacute phase, utilizing an AFO with a plantar-flexion stop [44]. The use of a plantar-flexion stop in the design of an ankle–foot orthosis results in increased dorsiflexion and knee flexion during the early stance phase of gait, which may lead to an increase in hip flexion. The ankle of the AFO-PS does not move into plantar flexion (Figure 4a). During gait with the AFO-PS, patients demonstrated a greater forward inclination of the pelvis upon initial contact compared to walking with shoes without an AFO. The use of an AFO-PS leads to a flexed alignment of the lower limb and a forward tilt of the pelvis [45]. The AFO-PS has been shown to improve dorsiflexion during the swing and early stance phases; however, it also results in a greater external knee-flexion moment during the loading phase compared to walking with shoes only and able-bodied control participants [46]. This increased acceleration into knee flexion may reduce knee hyperextension, but it can also cause instability in individuals with quadriceps weakness [45].

### 3.7. Hinged AFOs (HAFOs)

Ankle–foot orthoses (AFOs) with hinges, commonly referred to as hinged AFOs (HAFOs), are utilized when some level of ankle mobility is desired while certain limitations are still necessary [38]. The most prevalent HAFO designs include the overlap, Gillette, and Oklahoma joints (Figure 4b). The overlap joint restricts plantar flexion by interlocking the foot and shank shells, and it is secured by means of a rivet. The Gillette joint, on the other hand, links the shank shell to the foot shell as a separate entity, enabling movement in both the plantar-flexion and dorsiflexion directions. The Oklahoma joint, similar to the Gillette joint, establishes a connection between the shank shell and the foot shell as separate components, thus creating a gap between the shank shell and the posterior aspect of the foot shell, thereby allowing plantar flexion until the two parts make contact [38]. However, plantar flexion can also be fully restricted by positioning the shells at a 90-degree angle without any intervening space [38].

Hinged ankle–foot orthoses can be utilized to incorporate an articulating ankle joint, enabling dorsiflexion in the sagittal plane during stance while prohibiting plantar flexion during swing, as well as featuring support straps at the proximal tibia and, occasionally, at the ankle and forefoot [47]. These HAFOs may be equipped with a posterior strap to restrict the range of dorsiflexion [48]. It is important to note that they should not be employed by individuals with significant mediolateral instability of the ankle and are more appropriate for patients with adequate control over their knee joints [16,49,50].

### 3.8. Patellar-Tendon-Bearing AFO (PTB-AFO)

Patellar-tendon-bearing ankle–foot orthoses (PTB AFOs) differ from other types of plantar-ankle orthosis in that they include an additional anterior shell to assist in weight bearing via the patellar tendon [51]. This results in a reduction in the weight on the ankle, heel, and sole, potentially leading to a decrease in pain in these regions (Figure 4c) [51,52]. These orthoses are employed in situations that necessitate a reduction in pressure on the foot, such ulcers, calcanectomy, plantar skin grafts, severe ankle trauma/foot injuries, and fractures [38]. In essence, the brace was designed to shift the weight-bearing loads from the tibia, fibula, and foot bones to the lateral uprights [51]. The application of PTB AFOs can decrease the overall maximum plantar pressure on the foot; however, it may result in an increase in localized plantar pressure in the forefoot [53]. An excessive elevation of the heel reduces the contact area, thereby exacerbating the focal pressure on the forefoot. In general, a combination of the maximization of heel clearance and the restriction of ankle-joint movement appears to be the most effective means of reducing plantar pressure [51].

### 3.9. AFOs with Oil Dampers (AFO-ODs)

**Figure 5:**
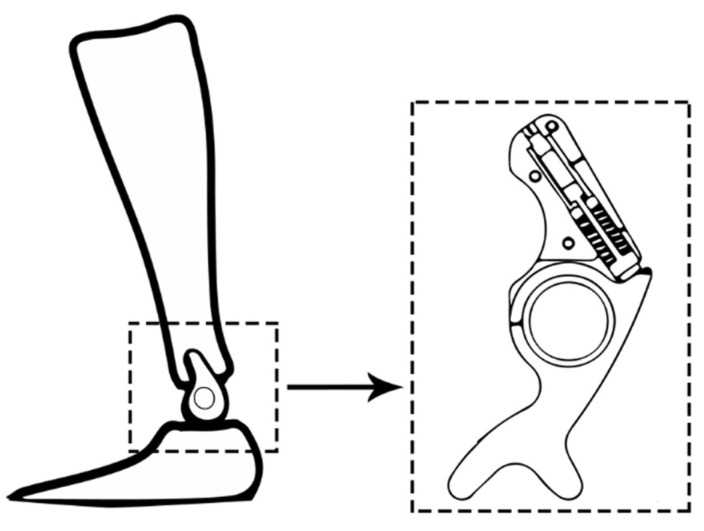
AFO with an oil damper [55]; with permission from Elsevier (License Number: 5513260140134).

The ankle–foot orthosis with oil damper (AFO-OD) was developed to aid heel-rocker function [54,55]. It features a functional unit (the oil damper) positioned on the lateral side of the ankle joint [56]. This unit contains a compact hydraulic cylinder, which can offer resistance against plantar flexion as required (Figure 5). During initial contact, when the ankle joint undergoes plantar flexion, the piston rod is pushed upward into the cylinder, which is filled with oil, resulting in resistance. Subsequently, upon the completion of plantar-flexion motion, a spring returns the piston to its initial position. The resistance of the oil damper can be easily modified by adjusting a screw [57].

### 3.10. Pneumatic Harvested AFOs (PhAFOs)

**Figure 6:**
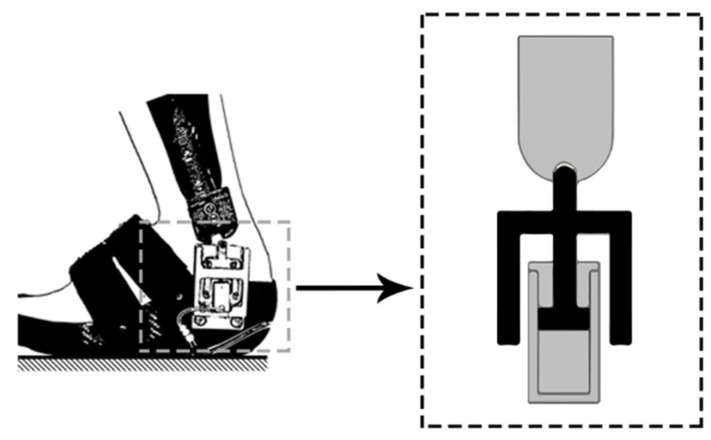
Pneumatic harvested AFO [59]; with permission from Springer Nature (Creative Commons license, http://creativecommons.org/licenses/by/4.0/).

Chin et al. [58,59] presented the concept of pneumatic harvested ankle–foot orthoses (PhAFOs), which are composed of two components fabricated from carbon-composite laminate: the tibial upright and the footplate. The sole of the device includes a bellow pump, an actuator, two check valves, a pressure-release valve, and a cam-lock mechanism attached on the lateral side of the PhAFO (Figure 6). The actuator in the PhAFO is composed of a roller follower, a linear cylinder, and a guide rail. During heel contact, compressed air is expelled from the cylinder into the atmosphere via the release valve.

The operation of the PhAFO involved a spring in the cylinder returning to its initial position, which then unlocked the cam and facilitated plantar flexion of the foot. During the stance phase, the bellow pump compressed and the release valve closed, leading to the extension of the roller follower, allowing free dorsiflexion due to the cam’s design. The cam mechanism, activated by the roller follower, ensured the absence of toe drag during the swing phase, as demonstrated in a gait-analysis study performed on a healthy individual wearing a shoe on one foot and a PhAFO on the other. The results of the kinetic and kinematic data analysis indicated that the toe had adequate clearance during the swing phase, although excessive dorsiflexion was observed during the mid-swing [42].

### 3.11. Short-Leg AFOs

The utilization of this particular type of ankle–foot orthosis is simple and convenient in terms of fitting it into footwear, and it is comparatively lightweight. Short-leg AFOs provide remarkable control over the foot and are considered to be an appropriate option for individuals suffering from flat feet. These devices maintain the foot in a perpendicular orientation with respect to the leg and, additionally, they can effectively counter the inward rotation of the foot, which is frequently observed in stroke patients with drop-foot [27]. One drawback of the fixed-hinge AFO prescribed for drop-foot is that it restricts plantar flexion and dorsiflexion, leading to an unnatural gait compared to other AFOs. Additionally, its shorter length makes it less suitable for taller individuals [27].

### 3.12. Energy-Return AFOs

The implementation of this type of ankle–foot orthosis, characterized by the incorporation of natural flexibility for enhancing dorsiflexion, has proven to be an effective solution. This AFO, often made from lightweight carbon-graphite materials, offers exceptional control without adding substantial weight. Clinical studies have demonstrated that the utilization of this AFO in individuals with hemiparetic stroke resulted in a 20% increase in walking speed and a 12% reduction in the energy cost per meter, as measured by oxygen consumption, when compared to unassisted walking [60]. In a separate study [61], the utilization of this type of AFO was shown to furnish support throughout the entire stance phase and enhance energy return during the third rocker phase of gait in a population consisting mainly of individuals with spina bifida. Furthermore, the spring element in the AFO contributed to a gait that was more in line with physiological principles.

A study published in 2008 [62] found that for individuals with spina bifida, the utilization of an energy-return ankle–foot orthosis resulted in more physiological ankle and knee kinematics and subsequently demonstrated a functional improvement in comparison to a more conventional orthotic device. However, the study also found that the kinetics and kinematics during the stance phase were significantly influenced by the alignment of the orthosis with the patients’ footwear. While these types of AFO possess numerous benefits, they may not be suitable for all individuals. Individuals with very large calf muscles or those who possess a naturally long stride, such as tall individuals, may encounter difficulties while utilizing these devices. Additionally, patients with spasticity or tight Achilles tendons may not find these AFOs to be optimal for their condition [63].

### 3.13. Three-Dimensionally Printed AFOs

Recently, there have been several attempts to produce an ankle–foot orthosis through the use of three-dimensional (3D) printing technology. These 3D-printed AFOs have the advantage of being easier to manufacture, with less skill and effort required, as well as being more easily replicable, compared to traditionally manufactured orthoses made through the molding of thermoplastic materials [64]. The repetition of AFO production is facilitated as the 3D-modeling file of the design is retained once. Furthermore, if an automated software program for orthotic design is established utilizing the pre-programmed orthotic-template design, the production of the AFO is simplified and can be personalized by patients themselves [64].

In recent years, several studies have been conducted to assess the viability of using 3D-printing technology to produce AFOs. These studies have focused on determining the functional properties of AFOs based on physical features, such as bending or rotational stiffness, and other material characteristics. Out of these studies, two replicated the design of a posterior-leaf-spring AFO [65,66], one replicated the design characteristics of a prefabricated carbon-fiber AFO [67], and six developed novel AFO designs [64,68,69,70,71,72]. The innovative concepts comprised the elaboration of a computer-aided, modeled, parameterized ankle–foot orthosis [68,69].

One investigation resulted in the production of a segmented ankle–foot orthosis, composed of 3D-printed foot and calf components, as well as a central, interchangeable carbon-fiber spring [71]. Another study incorporated a 3D-printed component with gas springs and commercially available bearings to yield an AFO with adjustable stiffness [70]. Other design configurations comprised ankle–foot orthoses that incorporated 3D-printing technology in the creation of 3-mm calf and foot sections connected by two carbon-fiber rods [72]. Additionally, other 3D-printed devices were developed for the support of the ankle and foot and were secured using laces [73].

The only investigation that did not result in the creation of a dynamic passive ankle–foot orthosis utilized 3D printing to fabricate a solid AFO, although no evaluation tests were conducted on this device [74]. A patient-satisfaction survey was conducted in another study [64] to compare the usage of a traditional AFO with that of a 3D-printed AFO. In regards to weight and usability, findings indicate that participants express a higher degree of satisfaction with 3D-printed AFOs. The conventional AFO was deemed challenging to wear due to its thickness [75]. Furthermore, studies have demonstrated that 3D-printed ankle–foot orthoses provide greater comfort during use [66] and elicit positive feedback regarding gait patterns following one hour of walking [68].

### 3.14. SMA-Based AFOs

Shape-memory alloys (SMAs) have been suggested as a potential solution to the issues associated with conventional ankle–foot orthoses, which are typically characterized by their weight, bulkiness, and limited functionality. The superiority of SMA-based AFOs is demonstrated by their lighter weight and more streamlined design, due to their higher power-to-weight ratio [76]. A study conducted by a researcher incorporated the use of superelastic wires and the installation of 14 plastic pulleys using screws and spacers [77].

**Figure 7:**
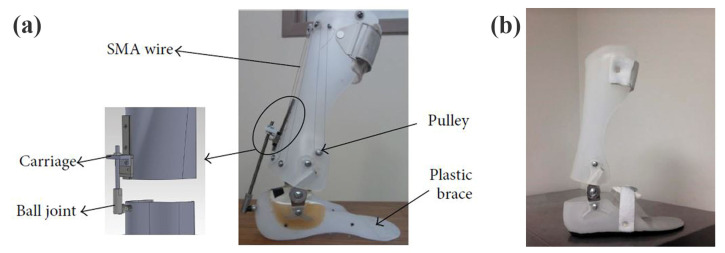
Images of SMA-based AFOs. (**a**) Superelastic-wire-based AFO [78]; with permission from Hindawi (Creative Commons license, http://creativecommons.org/licenses/by/4.0/). (**b**) Spring-based AFO [80]; with permission from MDPI (Creative Commons license, http://creativecommons.org/licenses/by/4.0/).

Another study employed a different approach, wherein the superelastic wires were affixed to the brace at one end and connected to a carriage at the other, as illustrated in Figure 7 [78]. Additionally, two novel adaptive solutions for the AFO mechanism based on SMA technology were proposed by other researchers, with the aim of modifying the stiffness in bending and torsion [79]. The first design concept involves altering the inner diameter and length of a superelastic rod in response to different controlled axial loads, thereby enabling the provision of variable torsional stiffness. The second design concept for SMA-based adaptive solutions for the AFO mechanism involved controlling the bending stiffness by adjusting the position of a slider in relation to the active length of a superelastic hinge. Furthermore, another study produced an AFO device that encompasses two superelastic SMA springs, a two-part brace, and two hinges, with an internal hole for mounting the springs, as illustrated in Figure 7 [80].

The stiffness profile of the ankle was found to resemble that of natural walking when NiTi springs were employed, and the shape-memory alloy (SMA) AFOs were capable of fulfilling the torque-angle specifications of the ankle-support device. Nonetheless, SMA-based AFOs present several limitations, including a low efficiency, of approximately 10%, and a limited bandwidth. Furthermore, the deflection of the SMA element occurs within a narrow temperature range, resulting in challenges in regulating partial contractions [81]. Table 1 presents a detailed summary of the passive articulated and non-articulated ankle–foot-orthosis types in terms of the control element, moment mechanism, features, and limitations.

### 3.15. Clinical Trials on AFOs

Kluding et al. [82] conducted a study examining the utility of ankle–foot orthoses (AFOs), using 197 stroke patients walking at normal and fast speeds. The study revealed that significant improvements in comfortable and fast walking speeds (0.18 m/s) were observed [82]. In a separate investigation, De Paula et al. [83] explored the impact of various types of AFO on the mobility and dynamic balance of 50 stroke patients. The study demonstrated that the use of AFOs led to better mobility and improved balance, as evaluated through the Timed Up and Go (TUG) test and Tinetti’s mobility scale.

Schwarze et al. [84] conducted a comparative study to evaluate the efficacy of laterally wedged insoles (LWI) and ankle–foot orthoses (AFOs) in 39 patients with medial-knee osteoarthritis. The maximum values of the knee-adduction moment (eKAM) and the Oxford Knee Score (OKS) were used as indices to assess the outcomes. The results demonstrated that both interventions led to significant improvements in the analyzed indices. Additionally, the use of AFO led to a substantial reduction in the maximum eKAM value, of 18%.

Bashir et al. [85] conducted a qualitative study aimed at exploring the perceptions of 15 male patients with peripheral artery disease regarding the use of ankle–foot orthoses (AFOs). The participants were divided into two groups: those who completed the AFO intervention and those who withdrew from it. The analysis of the semi-structured interviews revealed that the group that withdrew from the AFO intervention reported higher levels of physical discomfort. Conversely, the group that completed the intervention reported positive aspects, such as ease of standing and walking, as well as a reduction in pain.

In a study conducted by Miller et al. [86], the clinical and cost effectiveness of ankle–foot orthoses (AFOs) and functional electrical stimulation (FES) were compared over a period of 12 months in 85 individuals with multiple sclerosis and foot drop. The assessment of gait included various indices, such as the oxygen cost of walking, the Multiple Sclerosis Impact Scale-29, and the Modified Fatigue Impact Scale. The study findings revealed that the use of AFOs led to faster walking speeds in patients after 12 months, with significant improvements in various gait-assessment indices.

Raposo et al. [87] conducted a systematic review to assess the impact of ankle–foot orthoses (AFOs) on the gait of children diagnosed with spastic bilateral cerebral palsy (CP), using kinetic, kinematic, and functional outcomes. The study population consisted of 285 children with spastic bilateral CP, and the effects of five different types of AFO (solid, dynamic, hinged, ground-reaction, and posterior-leaf-spring) were analyzed. The analysis of the data showed significant differences in various gait parameters, such as walking speed, stride length, cadence, range of motion, ground-force reaction, joint moment, and functional score, when ankle–foot orthoses (AFOs) were used. This suggests that the utilization of AFOs by children diagnosed with spastic bilateral cerebral palsy (CP) may mitigate the effects of pathological gait, leading to consistent improvements in certain kinematic, kinetic, and spatial–temporal parameters.

Yeh et al. [88] developed an innovative energy-storage 3D-printed ankle–foot orthosis (ESP-AFO) and examined its impact on gait improvement in 12 stroke patients. The gait analysis was conducted using a motion-capture system, and the participants’ satisfaction and fatigue were also evaluated. The study findings revealed that the use of the ESP-AFOs led to a significant increase in bilateral gait velocity and stride length. Additionally, the ESP-AFO was shown to reverse drop-foot during the swing phase and to generate a greater ankle moment in the terminal stance, indicating that the newly developed custom-made ESP-AFO resulted in enhanced gait performance and higher satisfaction levels.

Fatone et al. [89] conducted a literature review to identify instruments utilized for evaluating the experience and outcomes of custom ankle–foot orthosis (AFO) care in individuals diagnosed with neurologic and traumatic conditions. The majority of the instruments utilized in the assessment of mobility included the 10-m-walk test, 6-min-walk test, Berg Balance Scale, Timed Up and Go, and Rivermead Mobility Index. These instruments demonstrated satisfactory reliability and validity and were regarded as viable options for developing quality measures pertaining to custom ankle–foot orthosis (AFO) care.

Moll et al. [90] conducted a study to evaluate the levels of activity and participation in daily life of 25 children diagnosed with cerebral palsy (CP), after the utilization of functional electrical stimulation (FES) and an ankle–foot orthosis (AFO). Each participant underwent twelve weeks of conventional treatment (AFO/adapted shoes), followed by 12 weeks of FES treatment, separated by a six-week washout phase. The researchers anticipated improvements in the level of bodily functions and structures, as well as activities, such as ankle kinematics and kinetics, which were measured using 3D gait analysis.

## 4. Conclusions

This literature review examined various currently available designs and types of passive ankle–foot orthosis (AFO). However, it was found that designing an AFO that is suitable for every patient remains a challenge, as the nature of disability varies among patients. Additionally, there is an ongoing challenge to develop an AFO that strikes a balance between ease of use, freedom of movement, high performance, and relatively low cost. Future studies should focus on identifying gaps in current knowledge and exploring emerging technologies to improve the efficacy, comfort, and cost-effectiveness of AFOs for individuals with gait disorders.

## Figures and Tables

**Table 1:** Detailed summary of the passive articulated and non-articulated ankle–foot-orthosis types.

Type	Control Element	Moment Mechanism	Features	Limitations
Metal AFO [28,29,30,31,32,33]	Springs/or hinges/or stops	Assistance/resistance or assistance/or locking	Customizable design: Metal AFOs can be customized to meet the specific needs of individual patients, including adjusting the joint positions and resistance levels to optimize gait patterns and improve functional outcomes. Durability: Metal AFOs are typically more durable and long-lasting than other types of AFOs. Provision of stability and support: Metal AFOs are designed to provide support and stability to the ankle and foot, which can improve balance, reduce falls, and prevent ankle injuries.	Heavy weight: Metal AFOs are typically heavier than other types of AFO, which can make them uncomfortable for some patients to wear for extended periods of time. Limited range of motion: Depending on their specific design of, metal AFOs may have a limited range of motion, which can affect gait patterns and functional outcomes. Higher cost: Metal AFOs are typically more expensive than other types of AFO, which can make them less accessible to some patients.
Plastic AFO [34,35,36]	One-way frictional clutch	Resistance	Light weight: Plastic AFOs are lightweight and more aesthetically appealing than other types of AFO. Low cost. Ease of fabrication: The fabrication process for plastic AFOs is relatively simple, which means they can be made quickly and easily. Noiselessness: Plastic AFOs do not produce noise when walking, which can be an advantage for patients who require quieter AFOs.	Limited adjustability: Plastic AFOs without hinges are less adjustable than hinged AFOs, which may limit their effectiveness in certain cases. Limited control of ankle movement in both plantar flexion and dorsiflexion directions, which may not be appropriate for all patients. Limited shock absorption. Skin irritation during prolonged use.
Posterior-leaf-spring AFO [37,38,39]	Plastic shell	Resistance	Light weight: The PLS AFOs are typically made of lightweight materials, making them more comfortable to wear for extended periods of time. Energy conservation: By assisting with plantar flexion during the swing phase of gait, PLS AFOs can help conserve energy and reduce fatigue during walking. Improved foot clearance: PLS AFOs provide improved foot clearance during the swing phase of gait, reducing the risk of tripping and falls. Ease of fit: PLS AFOs are relatively easy to fit and adjust, allowing a more personalized and comfortable fit.	Limited control: PLS AFOs provide limited control over ankle motion, particularly in the sagittal plane, which may not be sufficient for individuals with more severe gait abnormalities. Limited support: PLS AFOs provide minimal support to the ankle joint, which may not be sufficient for individuals with more significant weakness or instability. PLS AFOs may require frequent adjustments to maintain proper fit and alignment, which can be time-consuming and costly.
Ground-Reaction AFO [26,40]	Posterior leaf spring and a rigid footplate	Assistance	Provides good control of foot drop and stance phase stability. Reduces knee hyperextension. Improves ankle- and knee-joint alignment during weight bearing. Reduces energy expenditure during walking. Provides support for the medial longitudinal arch of the foot. Can be adjusted for different levels of activity.	Can be bulky and heavy. May limit ankle range of motion. May require shoes with high heel counters to provide proper fit and function. May require a break-in period for the patient to adapt to wearing the device. May be more expensive than traditional plastic AFOs.
Yamamoto Type I [41,42,43]	Two springs	Assistance	The level of assistance moments and the initial angle of the ankle can be adjusted easily by altering the lengths of the springs. Includes both dorsiflexion- and plantar flexion-assistance moments.	The presence of the posterior spring causes discomfort for hemiplegic patients. Limited range of motion, which can affect gait patterns and functional outcomes. Relatively heavy weight.
Yamamoto Type II [41,42,43]	One spring	Assistance	Does not incorporate a posterior spring. Allows relative freedom of movement in the ankle during dorsiflexion. Leads to a reduction in the knee-flexion moment and the absence of quick plantar flexion in the ankle.	May not provide sufficient support for patients with severe drop-foot or weak ankle muscles. Limited adjustability. May cause discomfort or pressure points on the shin or ankle bone.
Dorsiflexion-assistance-controlled spring AFO [42]	Dorsiflexion-assistance spring	Assistance	The DACS AFO provides adjustable dorsiflexion assistance with varying levels of support based on the spring selection. The DACS AFO allows more natural ankle-joint movement compared to rigid AFOs. Enhance walking speed through the reduction in knee hypertension and improvements in delayed progression in the hip joint.	The DACS AFO may not provide enough dorsiflexion assistance for severe drop-foot. The user may have difficulty with fitting and adjusting the device without assistance from a healthcare professional. The device may not be suitable for individuals with significant ankle instability or weakness, as it does not provide additional support. The spring mechanism may require regular maintenance and replacement.
Plantar-flexion-stop AFO [44,45,46]	Stops	Resistance	Effectively prevents plantar flexion during the stance phase, which helps to prevent falls and improve gait stability. Allows for normal ankle dorsiflexion and plantar flexion during the swing phase. Can be used in combination with other orthotic devices, such as knee-ankle–foot orthoses (KAFOs), to provide additional stability and support.	Limited ankle range of motion: AFO-PS devices prevent the ankle joint from moving beyond a certain point. While this can be helpful for preventing foot drop, it can also limit ankle movement, which may affect walking efficiency and balance. Discomfort and skin irritation: AFO-PS devices can cause discomfort and skin irritation, especially if they are worn for long periods of time. The proper fitting and adjustment of the device can help to reduce these issues, but they can still pose problems for some patients. Compliance: AFO-PS devices only work if they are worn consistently. Some patients may have difficulty complying with the device due to discomfort, difficulty putting it on or taking it off, or other issues. This can limit the effectiveness of the device.
Hinged AFO [16,38,47,48,49,50]	Hinges	Resistance or assistance	Improved ankle mobility: Hinged AFOs allow some ankle movement, which can improve walking efficiency and balance. This is especially beneficial for patients with mild-to-moderate ankle dysfunction who need some support but do not require complete immobilization of the joint. Compared to rigid AFOs, hinged AFOs can be more comfortable to wear, as they allow some movement and can be designed to fit more closely to the shape of the foot and ankle. This can reduce discomfort and skin irritation, especially when the device is worn for long periods of time. Customizable support: Hinged AFOs can be designed with varying degrees of support, depending on the needs of the patient. The hinges can be adjusted to limit or increase ankle movement, and the amount of support can be customized to provide the appropriate level of stability and control.	Limited ankle range of motion: While hinged AFOs are designed to allow some ankle movement, they still restrict the joint to some degree. This can affect walking efficiency and balance, especially in patients with severe ankle dysfunction. May require additional padding or socks to prevent chafing or blisters. May limit the range of motion in certain directions. Should not be employed by individuals with significant mediolateral instability of the ankle and are more appropriate for patients with adequate control over their knee joints. Maintenance: Hinged AFOs require regular maintenance to ensure that they continue to function properly. This can include replacing worn-out components, adjusting the fit of the device, and keeping the device clean and dry.
Patellar-tendon-bearing AFO [38,51,52,53]	Rigid frame	Resistance	Improved weight bearing: PTB-AFOs are designed to distribute weight away from the foot and ankle and onto the patellar tendon, which can be helpful for patients who have difficulty in bearing weight on the foot due to conditions such as foot ulcers or peripheral neuropathy. Enhanced proprioception: PTB-AFOs provide a high level of contact with the leg, which can enhance proprioception and improve balance and coordination. Employed in situations that necessitate a reduction in pressure on the foot, such as ulcers, calcanectomy, plantar skin grafts, severe ankle trauma/foot injuries, and fractures.	Restricted ankle motion: PTB-AFOs are designed to restrict ankle motion, which can affect walking efficiency and balance, especially in patients with severe ankle dysfunction. In addition, the use of PTB-AFOs significantly reduces ankle dorsiflexion and plantar-flexion range of motion in the gait of patients with peripheral neuropathy. Although the application of PTB AFOs can decrease the overall maximum plantar pressure on the foot, it may result in an increase in localized plantar pressure on the forefoot. The excessive elevation of the heel reduces the contact area, thereby exacerbating the focal pressure on the forefoot.
AFO with an oil damper [54,55,56,57]	Hydraulic damper	Resistance/assistance	Improved gait stability: The AFO-OD provides improved gait stability, especially during the swing phase, as the oil damper controls plantar flexion and prevents foot-slap. Comfort: The use of an oil damper can provide more comfortable support than other AFOs, as it reduces the risk of pressure sores and skin irritation. Reduced energy expenditure: The AFO-OD reduces the energy expenditure required for ambulation, as it controls plantar flexion and allows more efficient gait patterns. Adjustable resistance: The resistance of the oil damper can be adjusted to meet the specific needs of the user, allowing customization.	Cost: The AFO-OD can be more expensive than other AFOs due to the added technology and materials required for the oil damper. Maintenance: The oil damper requires regular maintenance, including oil changes and the potential replacement of worn or damaged components. Bulky appearance: The oil damper can make the AFO bulkier in appearance compared to other AFOs. Limited ankle range of motion: The oil damper restricts the ankle’s range of motion, which may not be suitable for certain individuals with specific needs.
Pneumatic harvested AFO [48,58,59]	Pneumatic cylinder	Resistance/assistance	Adjustable support: The use of air bladders in PhAFOs allows adjustable support to be provided to the ankle joint, which can be customized based on the patient’s needs and the stage of their rehabilitation. This adjustability can be particularly beneficial in patients with dynamic ankle instability or varying levels of ankle dorsiflexion or plantar flexion. Improved walking efficiency and reduced risk of falls: PhAFOs are designed to assist with ankle plantar flexion during the stance phase of gait, which can improve walking efficiency and reduce the energy required for ambulation. Cost-effectiveness: PhAFOs can be cost-effective alternatives to other types of AFO, as they do not require custom casting or fabrication. Furthermore, PhAFOs are more cost-effective than traditional carbon-fiber AFOs, with similar levels of patient satisfaction and clinical outcomes.	Limited ankle-dorsiflexion control: PhAFOs primarily provide support for ankle plantar flexion and may not be as effective at controlling ankle dorsiflexion. Patients with significant dorsiflexion weakness or spasticity may require additional ankle-dorsiflexion support from other types of AFO. Limited ankle stability: PhAFOs may not provide the same level of stability to the ankle joint as other types of AFO, such as rigid AFOs. This can be a concern for patients with significant ankle instability or weakness. Air leakage: The air bladders used in PhAFOs may be susceptible to air leakage, which can reduce the effectiveness of the orthosis and create the need for frequent adjustments or replacements. Comfort: While PhAFOs are generally lightweight and low profile, some patients may find them less comfortable than other types of AFOs due to the pressure from the air bladders.
Short-leg AFO [27]	Hinges or rigid shell	Assistance	Light weight: The SL-AFO is lightweight and can be worn for long periods without causing any discomfort. Low profile: The orthosis is designed to be low-profile, which means that it can be easily concealed under clothing. Cost-effectiveness: SL-AFO is relatively cheaper compared to other types of orthoses. Comfort: SL-AFO is designed to fit the shape of the patient’s foot and ankle, which makes it more comfortable to wear. Ease of adjustment: The SL-AFO is easy to adjust, which means that it can be customized to meet the specific needs of the patient.	Limited functionality: The SL-AFO has limited functionality, which means that it is not suitable for patients with severe lower-limb pathologies. Limited control: The SL-AFO provides limited control of ankle motion, which may not be suitable for patients who require more support. Limited durability: The SL-AFO may not be as durable as other types of orthosis and may need to be replaced more frequently. Its shorter length makes it less suitable for taller individuals.
Energy-return AFO [60,61,62,63]	Spring-like mechanism	Resistance/assistance	Improved gait efficiency: Energy-return AFOs are designed to store and return energy during the gait cycle, which can improve gait efficiency and reduce energy expenditure. Increased mobility: Energy-return AFOs may allow more natural and efficient gait patterns, which can increase mobility and help patients to perform activities of daily living with less effort. Reduced impact forces: Energy-return AFOs can reduce the impact forces on the foot and ankle during gait, which can be beneficial for patients with osteoarthritis, plantar fasciitis, or other foot and ankle conditions. Light weight and comfort: Energy-return AFOs are typically lightweight and low-profile, which can make them more comfortable to wear than other types of AFO.	Cost: Energy-return AFOs can be more expensive than other types of AFO due to their advanced materials and design. Durability: Energy-return AFOs may not be as durable as other types of AFO, particularly those made from more rigid materials. This can be a concern for patients who require long-term use of their AFO. Specific indications: ER-AFOs may not be suitable for all individuals with foot and ankle pathologies, and should be prescribed on a case-by-case basis. Individuals with very large calf muscles or those who possess a naturally long stride, such as tall individuals, may encounter difficulties while utilizing these devices. Additionally, patients with spasticity or tight Achilles tendons may not find these AFOs to be optimal for their condition.
Three-dimensionally printed AFO [64,65,66,67,68,69,70,71,72,73,74]	Design-based element	Resistance/or assistance/or locking	Customization: Three-dimensionally printed AFOs can be precisely customized to fit the patient’s foot and ankle anatomy, which can improve comfort and reduce pressure points. Light weight: Three-dimensionally printed AFOs are typically lightweight and low-profile, which can make them more comfortable to wear than other types of AFO. Material options: Three-dimensional printing allows a wide range of material options, including flexible materials, which can provide better shock absorption and reduced pressure points. Rapid prototyping: Three-dimensional printing allows rapid prototyping and design changes, which can reduce the time and cost involved in developing custom AFOs. Cost-effectiveness: In some cases, 3D-printed AFOs may be more cost-effective than other types of custom AFO, particularly when considering the time and labor involved in traditional manufacturing processes. Reduced waste: Three-dimensional printing can reduce waste by using only the materials needed to produce the AFO, which can be more environmentally friendly than traditional manufacturing methods.	Customization limitations: While 3D printing allows the precise customization of the AFO, it may not be suitable for patients with more complex foot and ankle conditions or unusual anatomies. In these cases, traditional AFO-manufacturing processes may be necessary to ensure adequate support and functionality. Production time: Three-dimensional printing can be a time-consuming process, particularly for complex or highly customized AFOs. This can result in longer waiting times for patients who require an AFO quickly. Equipment limitations: Not all orthotic facilities necessarily have access to 3D-printing equipment, which can limit the availability of 3D-printed AFOs for some patients. Cost: While 3D printing can be cost-effective for some AFOs, the initial investment in 3D-printing equipment and software can be high. This may make 3D-printed AFOs less cost-effective for facilities with lower patient volumes.
SMA-based AFO [76,77,78,79,80,81]	SMA element	Resistance/assistance	Light weight: SMA-based AFOs are typically lighter and less bulky than other types of AFO, which can make them more comfortable for patients to wear. Energy efficiency: SMA-based AFOs can store and release energy, which can help reduce the amount of energy required to walk or run. This can be particularly beneficial for patients with weak or paralyzed muscles. Adjustable: SMA-based AFOs can be programmed to adjust the level of support provided based on the patient’s activity level or other factors, which can improve comfort and function. Customizability: SMA-based AFOs can be customized to fit the patient’s specific foot and ankle anatomy, which can improve comfort and reduce pressure points. Reduced maintenance: SMA-based AFOs have fewer moving parts than other types of AFO, which can reduce the need for maintenance and repairs.	Cost: SMA-based AFOs may be more expensive than other types of AFO, which can be a consideration for some patients and healthcare providers. Limited availability: SMA-based AFOs may not be widely available in all areas, as they require specialized training and equipment to manufacture and fit. Limited durability: SMA wires have limited lifespans and can lose their effectiveness over time due to metal fatigue or other forms of wear and tear. Additionally, SMA-based AFOs can be sensitive to temperature and can lose their shape-memory properties when exposed to high temperatures.

## Data Availability

Data are available on request from the corresponding author.
